# COVID-19 and Breast Cancer Diagnosis in Brazil: An Analogy to the Sinking of the Titanic

**DOI:** 10.1200/GO.23.00108

**Published:** 2023-07-10

**Authors:** Ruffo Freitas-Junior, Aline Ferreira Bandeira de Melo Rocha, Henrique Lima Couto, Jordana de Faria Bessa, Linei Augusta Brolini Dellê Urban

**Affiliations:** Ruffo Freitas-Junior, MD, PhD, Mastology Program, Teaching Hospital, Federal University of Goiás, Goiânia, GO, Brazil, Araújo Jorge Hospital, Goiás Anticancer Association, Goiânia, GO, Brazil; Aline Ferreira Bandeira de Melo Rocha, MD, School of Medicine, Federal University of Goiás, Goiânia, GO, Brazil; Henrique Lima Couto, MD, PhD, Redimama-Redimasto Breast Unit, Belo Horizonte, MG, Brazil; Jordana de Faria Bessa, MD, Instituto D'Or de Pesquisa e Ensino, São Paulo, SP, Brazil; and Linei Augusta Brolini Dellê Urban, MD, Member of the National Committee on Mammography of the Brazilian College of Radiology, Curitiba, PR, Brazil

## TO THE EDITOR:

In a meticulously performed study published in *JCO Global Oncology*, Resende et al^1^ showed the impact of COVID-19 on patients with breast cancer at private health care institutes in Brazil. An increase was found in the number of cases of advanced disease at diagnosis, particularly in women older than 50 years, with an increase of 5.9% in stages III and IV.

We recently published a similar study on the effect of the pandemic on breast cancer staging and screening, albeit in women age 50 to 69 years seen within the Brazilian public health care system (Sistema Único de Saúde [SUS]).^2^ The present report highlights the current panorama and compares the findings in the public and private health care networks in Brazil.

The number of mammograms performed within the SUS decreased by 40% in 2020 and by 18% in 2021 in relation to 2019.^2^ Other reports have corroborated these data.^3,4^ After the restrictive measures of the pandemic were eased and vaccination implemented, the consequent contraction in SARS-CoV-2 infection rates and COVID-19–related mortality proved insufficient to restore breast cancer screening coverage to pre-COVID levels.^2,3^ Indeed, in 2021, an increase was found in the number of women who had not undergone screening for more than 3 years.^3^

During the pandemic, a reduction occurred in the number of cases of screening-diagnosed breast cancer^5^ while symptom-diagnosis increased.^3,5^ This finding is particularly concerning in a country in which breast cancer screening within the public health care system was already previously low,^2,6^ with a mean reported rate of 36.71% between 2013 and 2019.

Two studies conducted with data from the public health care system, one using secondary data for the entire country^2^ and the other with data for one single center,^5^ reported an increase of 10.7%-16.9% in stages III and IV during the pandemic, with the number of advanced cases at diagnosis exceeding the number of early diagnosed breast cancer cases among users of the SUS in Brazil as a whole.^2^

**FIG 1:**
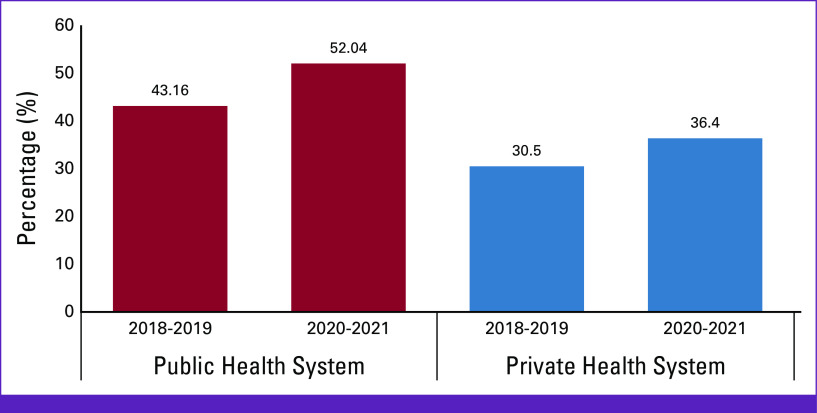
Comparison of the percentage (%) incidence of stages III and IV breast cancer in women older than 50 years of age in the public and private sectors in Brazil between 2018-2019 and 2020-2021.

We compared the data from our study with those from Resende et al to evaluate the rate of later stage diagnosis (stages III and IV) between the public and private health care services. Accordingly, we extended the age group in our study (50-69 years) to women older than 70 years and compared 2018-2019 with 2020-2021. Although there was an increase of 5.9% in later stage diagnosis in the private sector, the increase in the number of advanced cases in the public sector reached 8.88% between the two periods (Fig 1).

Before the pandemic, advanced stages at diagnosis were already more common among users of the SUS, 43.16% (unpublished data obtained by Oncology—Brazil Panel/DATASUS)^7^ compared with the private sector (30.50%).^2^ The pandemic potentiated this difference, with rates of 52.04% in the public sector versus 36.4% in the private sector (Fig 1). This highlights the increasing inequality in access to health care services in Brazil.

A previous study comparing the 10-year overall survival rate for patients with clinical stage III breast cancer between users of the two health care systems in Brazil showed rates of 55.6% in the private health care network and 39.6% in the SUS.^8^ Clearly, the effect of the pandemic on the prevalence of advanced stages will further compound the discrepancy in relation to breast cancer–related mortality between users of the different health care systems.

When the Titanic sank, less than a third of those on board survived, with the number of deaths being disproportionally high among third-class passengers compared with those in first class (74.78% *v* 37.84%).^9^ The situation in Brazil mirrors that catastrophe when data on breast cancer staging are compared between the public and private health care systems, highlighting the social inequalities in the country.

Promoting public policy interventions that will increase screening and patient access to health care services^6^ to higher levels than those seen before the pandemic is crucial and urgent. This is the only way in which the repercussions from the delayed diagnosis and treatment of patients with breast cancer could be reduced, thus avoiding the similar social discrepancies that occurred with the sinking of the Titanic.
